# A fast and robust method for the extraction and analysis of quaternary alkyl ammonium compounds from soil and sewage sludge

**DOI:** 10.1371/journal.pone.0237020

**Published:** 2020-08-04

**Authors:** Benjamin Justus Heyde, Anne Barthel, Jan Siemens, Ines Mulder

**Affiliations:** Institute of Soil Science and Soil Conservation, iFZ Research Center for BioSystems, Land Use and Nutrition, Justus-Liebig-Universität Giessen, Giessen, Germany; Centre for Ecology and Hydrology, UNITED KINGDOM

## Abstract

Alkyltrimethylammonium compounds (ATMACs), dialkyldimethylammonium compounds (DADMACs) and benzylalkyldimethylethylammonium compounds (BACs) are quaternary alkylammonium compounds (QAAC), which are released into the environment in large quantities after their use in cleaning agents and disinfectants. Despite their potential role as selective agents promoting resistance against QAACs as well as antibiotics, there is a lack of data for QAACs in soil due to the lack of sensitive analytical methods. Therefore, we present a robust and fast method for the extraction and quantification of concentrations of these compounds in soil and sewage sludge. The method is based on ultrasonic extraction (USE) with a mixture of acetonitrile and HCl followed by a solid phase extraction (SPE) cleaning step and a subsequent quantification of concentrations with high performance liquid chromatography with mass spectrometry (HPLC-MS/MS) in multi mass reaction mode (MRM). The proposed method is suitable for the quantification of ATMACs (chain length C-8 to C-16), BACs (C-8 to C-18) and DADMACs (C-8 to C-16). The achieved limits of quantification (LOQ) range from 0.1 μg kg^-1^ to 2.1 μg kg^-1^. The recovery rates of spiked soil samples for non-deuterated homologues were between 47% and 57%. The analysis of sewage sludge samples and soil samples revealed that BAC-C12 was the most abundant QAAC with concentrations up to 38600 μg kg^-1^ in sewage sludge and up to 81 μg kg^-1^ in a Mexican soil that was irrigated with wastewater. Overall, the presented methods open perspectives for effectively studying fate and effects of QAACs in soils.

## Introduction

Quaternary alkylammonium compounds (QAACs) are broadly used as disinfectants and surfactants in numerous applications. Although, QAACs and related resistance genes were found in several sediment and sewage sludge samples, the environmental fate and effects of QAACs are currently not well understood [1], also due to a lack of fast and reliable methods for extracting them from soils and analyzing their concentrations. The linear alkylammonium compounds alkyl trimethylammonium compounds (ATMACs), dialkyldimethylammonium compounds (DADMACs) and benzylalkyldimethylammonium compounds (BACs) are employed ubiquitously in industrial, hospital, agricultural and household chemicals [1]. All QAACs are made of a covalently bound, and therefore permanently positively charged, cationic nitrogen accounting for their hydrophilic and an alkyl chain for their hydrophobic properties. On the one hand, QAACs are biological degradable under aerobic conditions [2], on the other hand they exhibit biocidal properties depending on homologue. Generally speaking, the longer the alkyl chains and the less oxygen available, the more their biodegradability decreases and their toxicity increases [3, 4]

QAACs first appeared in the year 1935, when they were introduced as disinfectant agents by Domogk [5] and ever since, due to their unique surfactant as well as biocidal properties their production an use steadily increased. Starting in the 1990s they were heavily used as fabric softeners [6], especially QAACs of the DADMAC-type with C16 to C18 carbon chains. The use of these homologues was voluntarily phased out by the industry due to their poor degradability and replaced by more easily degradable esterquats [7]. Other QAAC homologues can be found in fabric softeners even today. Furthermore, QAACs are used as detergent in agriculture to clean machines and stables. The majority of the QAACs used for cleaning livestock buildings ends up in manure, which is used as fertilizer for agricultural fields, which represents one input pathway of QAACs to soils. Waste water treatment plants and sewage sludge represent other important sources of QAACs in the environment [2]. When wastewater is used for irrigation or sewage sludge for fertilization, QAACs are also released into the soil. Soil bacteria can adapt to QAAC, when QAAC concentrations remain sublethal [2, 1]. Since the genes encoding resistance against QAACs are often located on the same mobile genetic elements as antibiotic resistance [8], QAAC in soils can promote antibiotic resistance, which is one of the global challenges for human and animal health [9].

Despite their widespread application to soils with manure, wastewater or sewage sludge, the majority of the data available on QAACs in the environment deals with their concentrations in sewage sludges and wastewater [10, 11, 3]. Some data are available for QAACs in rivers and river sediments [12, 13], but only little information is available for soils. Gerike et al. [14] provided some data for concentrations of DADMACs in soil shortly after it was amended with sewage sludge. Similar to the Gerike et al. whose study focused on DADMACs, most other studies also address only one group of QAACs.

QAACs are analytically challenging due to their strong tendency to adsorb to surfaces. This can lead to a compound loss during the analytical process due to adherence to the equipment surfaces and, which might then also result in significant background concentrations caused by carry-over of the analytes between samples or between standards and samples. Since the chromatographic separation of the QAAC homologues is difficult, MS/MS detection in multiple reaction mode (MRM) is mostly used for differentiation of various homologues [13, 15–18].

Solid environmental samples, comparable to soil, like sediment and sewage sludge have been previously extracted with various methods: Steaming acidic methanol extraction [16, 12], ultrasonic extraction (USE) [19, 10] and accelerated solvent extraction [13]. Since Ruan et al [10] reached the lowest limits of detection (LOD) for sewage sludge samples with USE, this appeared the most promising method from which to develop an extraction method for soil and sewage sludge.

In summary, the surfactant nature of QAACs pose analytical challenges that leads to a lack of data. If included in multicompound-environmental screening methods, concentrations of QAACs tend to be underestimated. The development of a reliable method specific for the extraction and analysis of the group of QAACs from soils will contribute to completing our picture of the distribution of these components in the environment, thus promoting our comprehensive understanding of their environmental fate and effects. Hence, the goal of our work was to provide a stable, fast and, in terms of the needed extraction method, simple method for the group of QAACs.

## Experimental

### Reagents

The CAS numbers and suppliers of target analytes ATMAC-C8 to C16, BAC-C8 to C18, DADMAC-C8 to C16, Chlormequat and benzethonium that were used in the calibration and the internal standards BAC-C12-D7 and DADMAC-C10-D6 are shown in Table 1. A stock solution was prepared by mixing the reference substances with acetonitrile (AcN, ≥ 99.9%, HyperSolv, VWR, France) in large scale (100 mL), which was stored deep-frozen at -32°C. Working solutions, containing each of the 16 QAACs in the same concentration, were prepared in AcN one time before all analyses and stored at -32°C until analysis. aliquots were thawed at room temperature before analysis. Ultrapure water (MQ-water) that was used as eluent was made with Merck Milli-Q system (Millipore Merck, Darmstadt, Germany), methanol was purchased from VWR (≥ 99.9%, HyperSolv, France) and HCl from Merck (32%, p. A., Merck, Darmstadt, Germany). The extraction solvent containing (v/v) 99.9% AcN (≥ 99.9%, HyperSolv, VWR, France) and 0.1% HCl (Ph Eur, Merck, Darmstadt, Germany) was freshly prepared prior to every extraction.

**Table 1:** Supplier, CAS numbers and Mol. Masses of the target analytes and the internals standards.

Compound name	Abbreviation	CAS#	Mol. Mass
Alkyltrimethylammonium compounds (ATMACs)			
Octyltrimethylammonium bromide	ATMAC-C8*	2083-68-3	252
Decyltrimethylammonium chloride	ATMAC-C10*	10108-87-9	236
Dodecyltrimethylammonium chloride	ATMAC-C12*	112-00-5	264
Tetradecyltrimethylammonium chloride	ATMAC-C14*	4574-04-3	292
Hexadecyltrimethylammonium chloride	ATMAC-C16*	112-02-7	320
Dialkyldimethylammonium compounds (DADMACs)			
Dioctyldimethylammonium bromide	DADMAC-C8*	3026-69-5	350
Didecyldimethylammonium chloride	DADMAC-C10*	7173-51-5	362
Didodecyldimethylammonium chloride	DADMAC-C12*	3401-74-9	418
Ditetradecyldimethylammonium bromide	DADMAC-C14*	68105-02-2	519
Dihexadecyldimethylammonium bromide	DADMAC-C16*	70755-47-4	575
Didodecyldimethylammonium chloride deuterated	DADMAC-C10-D6^†^	-	368
Benzylalkyldimethylethylammonium compounds (BACs)			
Octylbenzyldimethylammonium chloride	BAC-C8*	959-55-7	284
Decylbenzyldimethylammonium chloride	BAC-C10*	965-32-2	312
Dodecylbenzyldimethylammonium chloride	BAC-C12*	139-07-1	340
Tetradecylbenzyldimethylammonium chloride	BAC-C14*	139-08-2	368
Hexadecylbenzyldimethylammonium chloride	BAC-C16*	122-18-9	396
Octadecylbenzyldimethylammonium chloride	BAC-C18*	122-19-0	424
Dodecylbenzyldimethylammonium chloride deuterated	BAC-C12-D7^†^	-	347
Chlormequat chloride	ChlM^‡^	999-81-5	158
Benzethonium chloride	BenzEth^‡^	121-54-0	448

*supplied by TCI, Eschborn, Germany

†supplied by HPC Standards (Cunnersdorf, Gemany)

^‡^supplied by Sigma-Aldrich, Steinheim, Germany.

For SPE the washing solution contained 1.5 mol L^-1^ HCl (32%, p. A., Merck, Darmstadt, Germany) in water and for column elution the solution was a mix of methanol (≥ 99.8%, VWR, Darmstadt, Germany) and 1.5 mol L^-1^ HCl (4:1, v/v).

The buffer solution for high performance liquid chromatography (HPLC) was prepared with formic acid (≥ 98%, Rotipuran, Carl Roth, Karlsruhe, Germany) and ammonium formiate (99.0%, Acros Organics, Thermo Fisher, Waltham, USA). Pentane (≥ 99%, Rotisolv) for spiking the soil was from Carl Roth (Karlsruhe, Germany).

### Instruments and materials

#### Extraction equipment

The evaporation unit (Synchor Polyvap) the associated 120 mL glass vessels, and the Pressurized Solvent Extractor (PSE) were from Büchi (SpeedExtractor, Flawil, Switzerland) and employed for sample extraction trials. Additionally, and ultrasonic bath (Super RK 225 H) from Sonorex (Berlin, Germany) was used. The following solid phase extraction (SPE) cartridges were tested during optimization: Chromabond CN, Chromabond C18 ec and Chromabond HLB (6 mL, 1000 mg, Machery Nagel, Dueren, Germany), and Oasis HLB (6 mL, 500 mg, Waters, Eschborn, Germany). Twenty mL amber glass extraction vials with PTFE lid were obtained from CS Chromatography (Langerwehe, Germany) and 2 mL safe-lock tubes from Eppendorf (Hamburg, Germany). Fifteen mL polypropylene centrifuge tubes for SPE were purchased from neoLab Migge (Heidelberg, Germany). The Rotanta 460R centrifuge was form Hettich (Tuttlingen, Germany) and the Orbital shaker KS-10 from Bühler (Bodenlshausen, Germany). Prior to the experiments, all of the used analytical flasks and vials were cleaned with AcN.

#### HPLC-MS/MS analysis

The analyses were performed with a Waters™ alliance 2690 separations module, which was equipped with an autosampler unit, a gradient pump, a column oven and a sample heater. The chromatographic device was connected via Peek capillary (0.18 mm ID) to a Waters™ triple quadrupole mass spectrometer (Micromass Quattro Micro). The final optimized method used a Waters XSelect CSH Phenyl-Hexyl-Column (130 Å, 150 mm length, 2.1 mm ID, 3.5 μm particle size) and a column guard of the same material. Further tested HPLC columns were zorbax RX-C8 (Agilent, 150 mm length 2.1 mm ID, 5 μm particle size) and Nucleodur π^2^ (Macherey Nagel, Düren, Germany, 250 mm length 3 mm ID, 5 μm particle size). The 2 mL clear glass vials for the extracts were purchased from neoLab Migge (Heidelberg, Germany).

### Procedures

#### Sample preparation

Luvisol samples were taken from a depth of 0 to 30 cm near Hungen in Northern Hesse, Germany, ten days after the application of sewage sludge for fertilization. A soil that was sampled from an adjacent field with similar soil properties was used for spiking with analytes, but this soil was not fertilized with sewage sludge for a minimum of ten years. Vertisol samples (0 to 30 cm depth) were taken from the Mezquital Valley in central Mexico. Those samples were irrigated with wastewater from Mexico City. Sewage sludge samples from wastewater treatment plants in northern Hesse were freeze-dried prior to extraction.

Soil and sewage sludge samples were collected in aluminum foil and deep-frozen at– 32°C, freeze-dried for ten days and sieved to < 2 mm afterwards. The sampling tools such as spade and bucket were cleaned with EtOH in between processing different samples.

For the recovery trial, soils that received no sewage sludge or wastewater were spiked with 1 μmol methanolic solutions of ATMAC-C12, ATMAC-C14, BAC-C12-D7, BAC-C16, DADMAC-C12 and DADMAC-C10-D6. To this end, 500 g of freeze-dried and sieved soil were covered overnight with a mixture of pentane and the QAACs solution while shaking continuously in and orbital shaker at 150 rpm with the rest of pentane removed by evaporation the following day. Unspiked control samples were treated in the same way without the addition of QAACs.

#### Extraction and sample clean up

The final extraction procedure for the target analytes was based on Xiang et al. [20], who used an ultrasonic extraction method for the extraction of three QAACs from vegetables. We placed 5 g of soil or sewage sludge in 20 mL amber glass vials and mixed it with 10 mL of the extraction solution (99.9% AcN / 0.1% HCl v/v). The capped vials were then shaken at 420 rpm for 10 min on an orbital shaker and placed in an ultrasonic bath for 10 min at room temperature. After centrifuging at 870 Relative Centrifugal Force (RCF) for 10 min. the extracts were decanted to evaporator vessels. These steps were repeated three times and the collected extract supernatants were evaporated to ≤ 1 mL. and, if necessary, filled up to 1 mL with the extraction solution. The extracts were transferred to safe-lock tubes and centrifuged at 17.000 RCF to remove suspended particles. After transferring the soil extracts to 1.5 mL clear HPLC glass vials they were deep-frozen until HPLC-MS/MS analysis.

Sewage sludge sample extracts required a clean-up step as they contained large amounts of dissolved and particulate organic matter. Therefore, a SPE was implemented according to the manufacturers application guide. Macherey & Nagel CN cartridges were conditioned with 12 mL MQ-water. The 1 ml extract volume from sewage sludge samples were transferred from the safe-lock tubes into 15 mL polypropylene tubes and diluted to 6 mL with MQ-water. Afterwards, the diluted extracts were percolated through the cartridges for 10 minutes. The cartridges were washed afterwards with 6 mL of MQ-water and twice with 1 mL of HCl and dried under vacuum for 30 s. The columns were eluted twice with 1 mL of methanol HCl solution. The samples were evaporated under a constant flow of nitrogen to ≤ 1 mL. and, if necessary, filled up to 1 mL with methanol and collected in HPLC vials for the analysis.

Extracts were stored at -32°C until HPLC-MS/MS analyses. In cases where the detected concentrations exceeded the calibration range, the extracts were diluted with the extraction solution to an expected concentration between 2.5 μg L^-1^ and 300 μg L^-1^.

**Fig 1:**
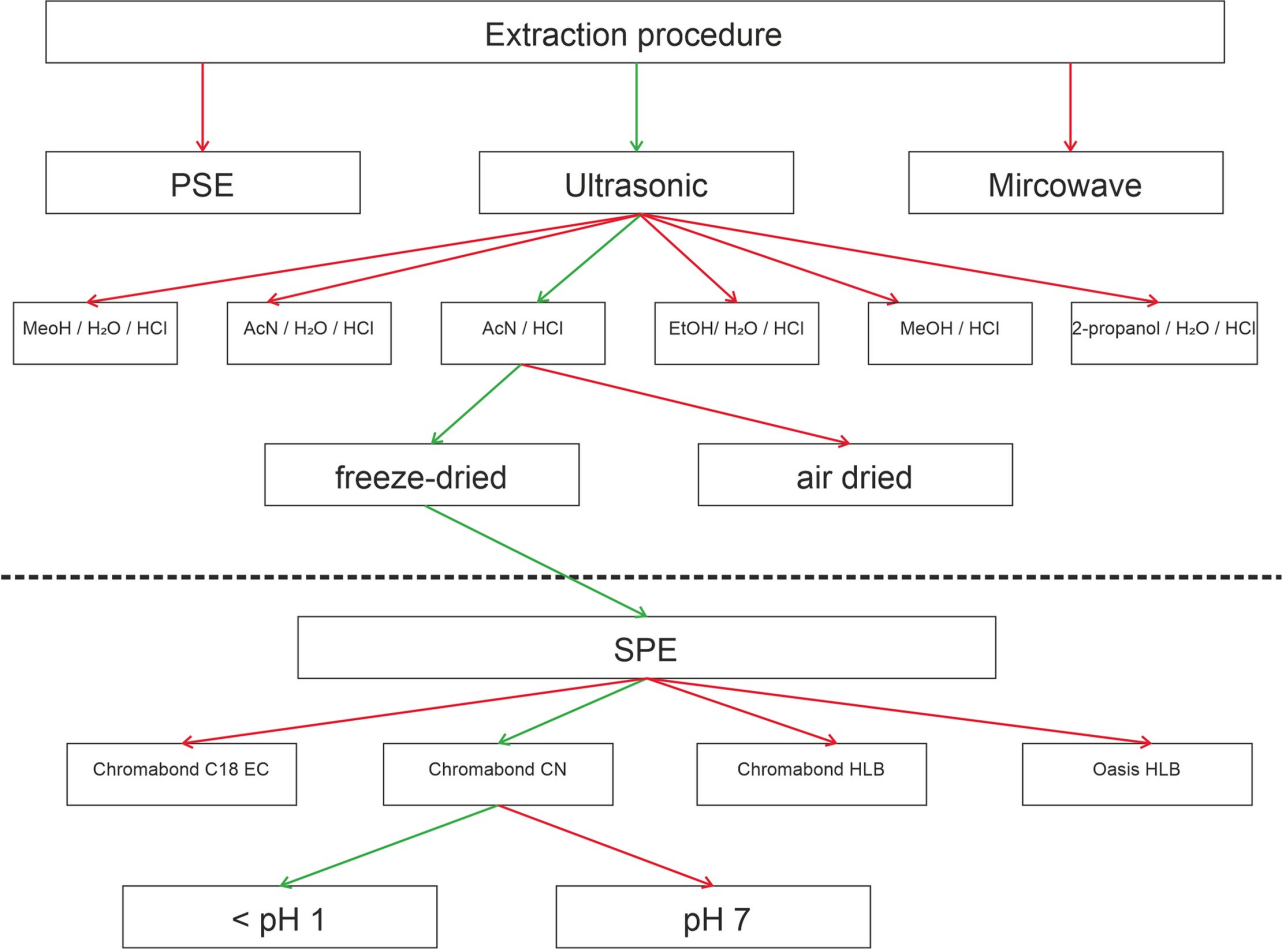
Schematic overview of the development of the extraction method. Green arrows indicate the chosen optimum procedure, red arrows represent dead ends with suboptimal results that were not further followed. The initial method development was performed for soil samples, and was subsequently also applied to sewage sludge samples.

Fig 1 summarizes the various extraction procedures and solvent mixtures that were tested for optimal extraction yield. PSE was performed with 0.5 g of soil at 120°C and 103 bar (3 cycles) and microwave extraction with 0.5 g of soil and 750 W for 30 min after 5 min preheating at 450 W. The centrifuging and evaporating procedures were performed analogously to the final method described above.

#### High performance liquid chromatography with mass spectrometry (HPLC-MS/MS)

**Fig 2:**
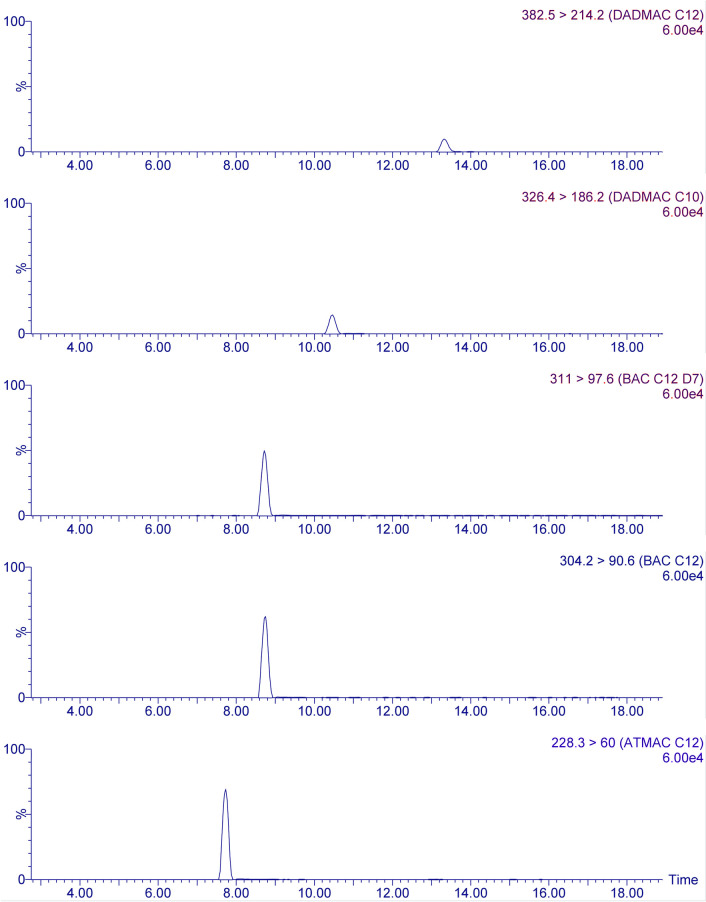
Chromatogram of DADMAC-C10, BAC-C12-D7, BAC-C12 and ATMAC-C12 in concentrations of 200 μg L^-1^ in AcN.

For HPLC-MS/MS measurements, 20 μL of the soil or sewage sludge extracts were injected into the system. A buffer solution of formic acid and ammonium formate mixed with AcN were used as liquid phase for the chromatography. As mobile phase (A) a buffer solution containing 50 mM formic acid and 10 mM ammonium formate (90%) mixed with AcN (10%) and as mobile phase (B) a mixture of the buffer solution of formic acid and ammonium formate (10%) and AcN (90%) were used. As shown in Table 2A both eluents were premixed 90% / 10% (v/v) to prevent precipitation of salt in the HPLC system. The flow rate was set to 0.25 mL min^-1^. After separation, the analytes were detected with tandem mass spectroscopy in MRM mode with argon (≥ 99.999%) as collision gas. Precursor and product ions as described by Ruan et al. [10] were used for ATMACs, BACs and DADMACs, those for chlormequat and benzethonium were determined in our lab. Based on very stable retention times of the analytes (variation < 12 seconds, see below), the specificity of the transition from the precursor ion to the product ion, and our excellent experience with spiked samples regarding the identification of the target analytes, we decided to maximize the sensitivity of our mass spectrometer by detecting 20 product ions instead of 40 ions. Detailed MS/MS settings are presented in Table 2B. Chromatograms of DADMAC-C10, BAC-C12-D7, BAC-C12 and ATMAC-C12 are displayed in Fig 2, chromatograms of all analytes and a total ion current chromatogram are provided in SI (S2 and S3 Figs).

**Table 2a:** Settings and Parameter for the HPLC as used to analyze QAACs.

Time	90% H_2_O + buffer 10% AcN (A)	10% H_2_O + buffer 90% AcN (B)	Flow
	[%]	[%]	[mL min^-1^]
0	100	0	0.25
3	25	75	0.25
14.5	0	100	0.25
21	0	100	0.25
21.01	100	0	0.25
26	100	0	0.25

Waters XSelect Phenyl Hexyl Column; 38°C column temperature; 20 μL injection; buffer = 50 mM formic acid + 10 mM ammonium formate.

**Table 2b:** Settings and Parameter for the MS/MS as used to analyze QAACs. Qualifier ion is given in brackets behind the quantifier ion.

	RT [min]	Precursor ion [m z^-1^]	Product ion [m z^-1^]	Collision [V]
ATMAC-C8	6.3*	172*	60* (57*)	25*
ATMAC-C10	7.0*	200^†^	60^†^ (57^†^)	25*
ATMAC-C12	7.6*	228^†^	60^†^ (57^†^)	25*
ATMAC-C14	8.4*	256^†^	60^†^ (57^†^)	27*
ATMAC-C16	9.3*	284^†^	60^†^ (57^†^)	27*
BAC-C8	7.2*	248^†^	91^†^ (58^†^)	23*
BAC-C10	7.8*	276^†^	91^†^ (58^†^)	23*
BAC-C12	8.6*	304^†^	91^†^ (58^†^)	25*
BAC-C14	9.6*	332^†^	91^†^ (58^†^)	25*
BAC-C16	10.8*	361^†^	91^†^ (58^†^)	27*
BAC-C18	12.4*	389^†^	91^†^ (58^†^)	29*
DADMAC-C8	8.4*	270^†^	158^†^ (57^†^)	25*
DADMAC-C10	10.3*	326^†^	186^†^ (57^†^)	29*
DADMAC-C12	13.1*	383^†^	214^†^ (57^†^)	32*
DADMAC-C14	16.5*	439^†^	242^†^ (57^†^)	35*
DADMAC-C16	20.5*	495^†^	270^†^ (57^†^)	39*
Chlormequat	1.6*	122^†^	58^†^ (63*)	33* (35*)
Benzethonium	8.9*	413^†^	91^†^ (320*)	50* (30*)
BAC-C12-D7	8.6*	311^†^	98*	37*
DADMAC-C10-D6	10.3*	332^†^	192*	41*

Cone voltage [V] = 43; Capillary voltage [kV] = 3.5; Source [°C] = 130; Desolvation °C [450]; Desolvation Gas N^2^ [L hr-1] = 600.

* determined in our lab

^†^ Ruan et al. [10].

#### Calibration and quantification

For quantification, an external eight-point calibration (2.5, 5.0, 10, 20, 40, 80, 160 and 300 μg L^-1^) was prepared in AcN before each analysis, with the working solution warmed to room temperature before use. As injection standard, a deuterated BAC-12 (BAC-12-D7) was added to every single extract and calibration sample immediately before sample injection. The concentration of the injection standard was consistently 100 μg L^-1^. Peak analysis was performed with Waters MassLynx 4.0, data evaluation and illustration with Systat Sigma Plot 12.0. In order to correct the analysis for instrumental drift, the quotient of the signal of the analyte and the injection standard was used for quantification.

The limit of Detection (LOD) *x_LOD_* and limit of quantification (LOQ) *x_LOQ_*

(μg kg^-1^) were calculated as shown below,

$$x_{LOD}=4\times \sigma$$ (1)

$$x_{LOQ}=10\times \sigma$$ (2)

with σ (as μg kg^-1^) denoting the standard deviation of the blank values (n = 12).

## Results and discussion

### Procedure optimization

In the following, different steps in our method development procedure are presented and discussed. The flow chart in Fig 1 offers a schematic guide through our optimization procedure. The initial method development was performed for soil samples, and was subsequently also applied to sewage sludge samples that required SPE cleanup.

#### Sample extraction

Acidified MeOH [15, 16, 19, 21] and AcN [22, 23] are the most commonly used solvents for the QAAC extraction of sludges, water and sediments. In our extraction trials we found that AcN with HCl worked best for soils (Table 3). Recovery rates of spiked QAACs extracted with USE and other extracting agents (EtOH, MQ-water, 2-Propanol, see Table 3) were all ≤ 10%. Mixtures of water and organic solvents had poor recoveries in our corresponding extraction tests, which is contradictory to Ferrer and Furlong [13], whose work showed that the extraction of BAC-C14 from sediment samples worked better with a mixture of organic solvent and water compared to pure organic solvents. PSE and Microwave extraction were tested as well, but QAAC recoveries were insufficient (Table 3).

**Table 3:** Extraction efficiency of spiked soil samples expressed as recovery, as well as precision, Limit of Detection (LOD), Limit of Quantification (LOQ) and blank value of ultrasonic extraction with AcN (99.9%) and HCl (0.1%) using 0.5 g of soil (n = 3).

	PSE A	PSE B	MWE	Ultrasonic extraction				
	Recovery			Recovery	Precision (SD)	LOD	LOQ	Blank values
	[%]			[%]	[%]	[μg kg^-1^]		
ATMAC-C12	< LOD	24	4	47	2.5	2.2	2.5	< LOD
ATMAC-C16	< LOD	< LOD	< LOD	51	1.3	3.9	6.0	< LOD
DADMA-C12	18	< LOD	< LOD	55	3.0	3.5	4.3	< LOD
DADMAC-C10 D6	< LOD	< LOD	< LOD	33	1.7	4.5	6.8	< LOD
BAC-C12 D7	< LOD	3	< LOD	43	1.8	2.9	3.3	< LOD
BAC-C16	< LOD	14	51	57	2.4	3.2	4.6	< LOD

The final optimal extraction method using USE and acidic (0.1% HCl) AcN provided recovery rates of the unlabeled QAACs between 47% (ATMAC-C12) and 57% (BAC-C16) with standard deviations ≤ 3% for replicate extractions (Table 4). Surprisingly, the extraction efficiencies for deuterated standards added were roughly 10% lower, possibly due to substitution of the H^2^ with H^1^ in the labeled BAC and DADMAC. The work by Davison et al. supports this hypothesis as they also observed deprotonation and substitution of deuterium for 5-hydroxyindole-4,6,7-d3-3-acetic-2,2-d2 acid within their labeled standard [24].

**Table 4:** Extraction methods that were tested for the highest recovery of QAAC from soil samples.

Extraction method	Extracting agent [%]
Microwave extraction (MWE)	AcN [60] / H_2_O [40]
Pressurized solvent extraction (PSE A)	AcN [60] / H_2_O [40]
Pressurized solvent extraction (PSE B)	MeOH [50] / H_2_O [50]
Ultrasonic extraction (1)	MeOH [99.9] / HCl [0.1]
Ultrasonic extraction (2)	MeOH [59.45] / H_2_O [39.45] / HCl [0.1]
Ultrasonic extraction (3)	AcN[59.45] / H_2_O [39.45] / HCl [0.1]
Ultrasonic extraction (4)	2-Propanol [59.45] / H_2_O [39.45] / HCl [0.1]
Ultrasonic extraction (5)	EtOH [59.45] / H_2_O [39.45] / HCl [0.1]
Ultrasonic extraction (6)	AcN [99.9] / HCl [0.1]

Importantly, for our extraction procedure we found it to be essential that freeze-dried samples were used. Recovery rates for air-dried samples were only half as high as for freeze-dried samples. Potential reasons for higher recoveries from freeze-dried samples than from air-dried samples are a better accessibility of sorbed QAACs in the fine powder of freeze-dried samples and the prevention of biodegradation that might occur during the air-drying procedure.

The extracts of sewage sludge consisted of two visible liquid phases, which also contained large concentrations of dissolved organic matter and suspended particles. Therefore, SPE was necessary for the sewage sludge extracts for sample cleanup. As it is clear from Fig 1, out of the four SPE cartridges tested by following its application guides and Östman et al. [25], only CN columns eluted with 1.5 mol L^-1^ HCl acidified MeOH showed satisfying recovery rates around 50% (further detail in S1 Fig). DADMACs with an alkyl chain longer than 12 C atoms had very low recovery rates around 20% (S1 Fig), compared to reference samples that were not subject to SPE, highlighting their stronger tendency to interact with the cartridge surface due to increasing hydrophobicity with increasing chain length [3]. Also, chlormequat is almost fully eliminated in this step, possibly because of the strong polarity of its chlorogroup. Another reason could be the specific interaction of the free electron pair of the CN group with the electro negative chlorgroup, since the CN cartridge mechanism is, according to providers catalogue, based on weakly hydrophobic interactions and specific interaction.

#### Chromatography

First chromatographic experiments were performed with three eluents (MeOH, water, acetic acid and ammonium acetate in iso-propanol) and a Zorbax RX-C8 analytical column (2.1 mm i.d. × 150 mm length, 5 μm, Agilent) as described by Ruan et al. [10]. We reduced the number of eluents to two and also used the aqueous phase rather than the organic phase to buffer the pH in order to prevent precipitation of salts. The organic and the aqueous phase were premixed (90% / 10%, v / v) with each other for the same reasons and also to reduce the risk of bubbles in the system.

We then tested three different HPLC columns, XSelect, Zorbax RX-C8 and Nucleodur π^2^. The best results were obtained with the XSelect column, with optimal separation and sharp peaks (Fig 2; S2 Fig). The final gradient program, which ends with a five minute re-equilibration step, is shown in Table 2a.

The pH values of the SPE extracts were very acid (below 1), hence NaOH was used to adjust the values to around pH 7. However, recovery rates were lower with adjusted pH values. We assume that the formation of salt crystals resulted from the reaction of dissolved Na^+^ and Cl^-^ (from the QAAC counterion). Coprecipitation phenomena of the QAACs such as the adsorption to or the occlusion in the NaCl crystals could have led to the reduced QAAC recovery.

We concluded, that in the light of the acid tolerance of the analytical column of choice, the lengthy procedure of pH adjustment was not necessary. Regarding to retention times, the peak analysis confirmed that the adjusted and non-adjusted samples were identical.

The negative effect of SPE on recoveries of DADMACs with longer alkylchains (> 12 Cs) did also occur in chromatography. Retention times increased due to the strong hydrophobic interaction of the alkylchains with the analytical column and peak broadening occurred. Therefore, we excluded DADMAC-C18 from the method. In contrast to the effect of the SPE, the small molecule chlormequat showed only little interaction with the XSelect column and was first to elute after 1.6 minutes (Table 2B).

### Method validation

#### Limit of detection and quantification

**Fig 3:**
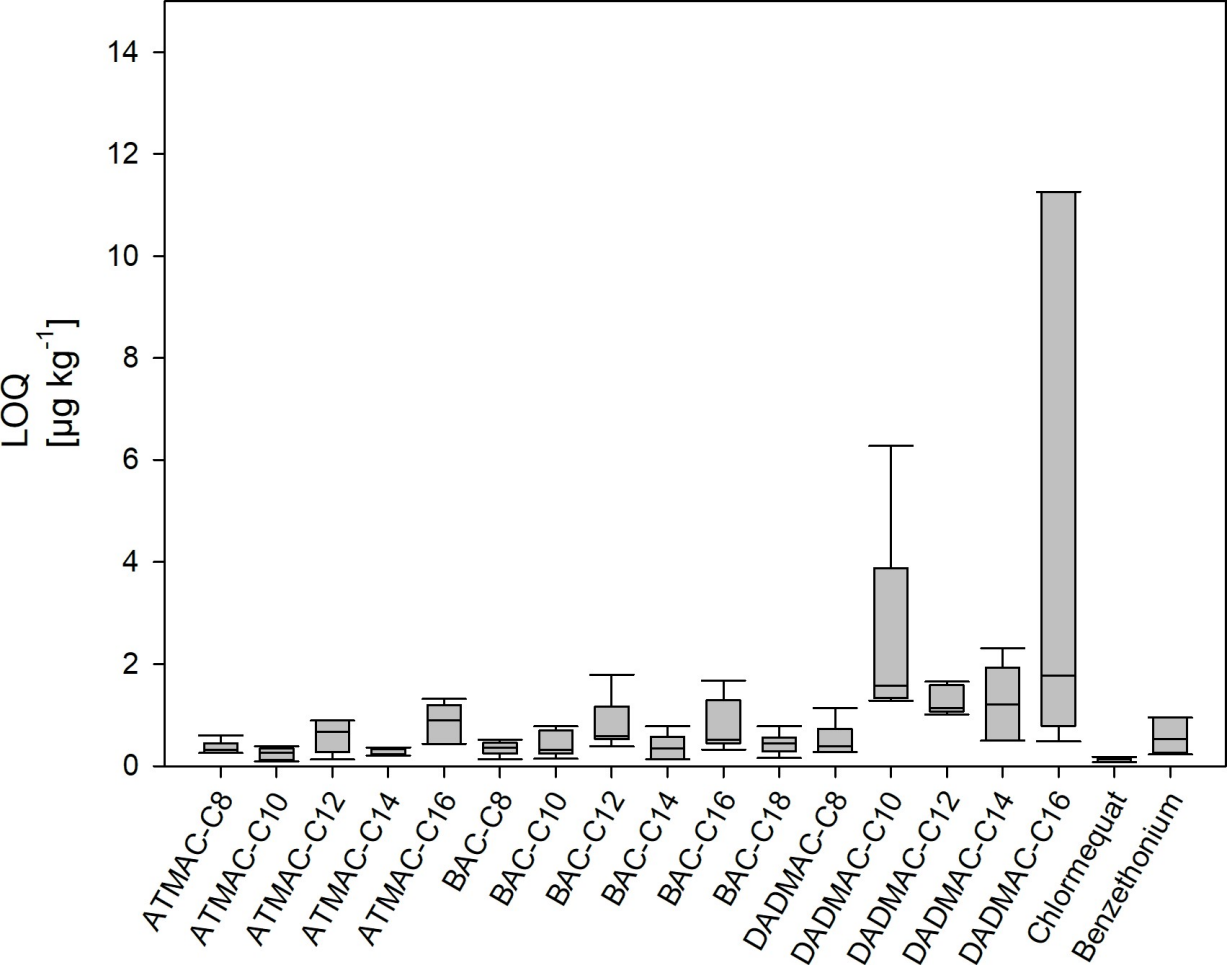
Limits of quantification of the HPLC-MS/MS method during two years of routine analyzes.

Table 5 lists the LODs and LOQs for all analytes. Additionally, LOQs determined over an extended period of time during routine operation are shown in Fig 3. From Fig 3 it is apparent, that the robustness of the method depends on the analytes. The best results were obtained for ATMACs in general and, in particular, for ATMAC-C10. In contrast, DADMACs were analytically challenging, especially those with alkyl chains longer than C12. LODs for the most common QAACs BAC-C12 and DADMAC-C10 were 0.4 μg kg^-1^ and 1.3 μg kg^-1^, respectively. In general, our achieved LODs were lower compared to methods for sewage sludge and sediment rivers as described by Merino et al., Bergé et al., Ruan et al. and Martinez-Carballo et al. [10, 11, 15, 26]. An overview of the studies and their LOQs compared to our work is given in Table 6.

**Table 5:** Limit of Detection (LOD) and Limit of Quantification (LOQ) and coefficient of determination (R^2^) of calibration from 2.5 μg L^-1^ to 300 μg L^-1^.

	LOD [μg kg^-1^]	LOQ [μg kg^-1^]	R^2^
ATMAC-C8	0.2	0.3	0.996
ATMAC-C10	0.1	0.2	0.997
ATMAC-C12	0.3	0.5	0.998
ATMAC-C14	0.1	0.3	0.999
ATMAC-C16	0.5	1.1	0.999
BAC-C8	0.2	0.3	0.996
BAC-C10	0.3	0.5	0.998
BAC-C12	0.4	0.8	0.999
BAC-C14	0.2	0.4	0.999
BAC-C16	0.4	0.9	0.999
BAC-C18	0.3	0.5	0.997
DADMAC-C8	0.3	0.5	0.998
DADMAC-C10	1.3	2.1	0.997
DADMAC-C12	0.7	1.4	0.999
DADMAC-C14	0.9	1.3	0.996
DADMAC-C16	0.8	1.7	0.997
Chlormequat	0.1	0.1	0.992
Benzethonium	0.2	0.4	0.999

**Table 6:** Limit of Quantification (LOQ) of our work compared to works with similar Matrices and their number of the analyzed homologues. Extraction methods were Acid-Induced Cloud-Point (ACPE), Solid Phase Extraction (SPE) an Ultrasonic Extracition (USE).

	Matrix	Extraction	Detection	LOQ [μg kg^-1^]	# homologues
Merino et al. [11]	Raw sewage	ACPE	LC—MS	40–70 *	3 ATMACS; 4 BACs; 3 DADMACs
Bergé et al. [26]	Biological sludge	SPE / QuEChERS	LC—MS/MS	20–40	2 BACs
Ruan et al. [10]	Sewage sludge	USE	LC—MS/MS	1.3–4.2	7 ATMAC; 6 BACs; 7 DADMACs
Martinez-Carballo et al. [15]	Sediment and Sludge	Soxhlet	LC—MS/MS	0.6–3	3 ATMAC; 4 BACs; 5 DADMACs
This work	Soil and sewage Sludge	USE	LC—MS/MS	0.1–2.1	5 ATMAC; 6 BACs; 5 DADMACs

* LOQ were not shown by this work, so their LOD are displayed here.

#### Matrix effect

**Fig 4:**
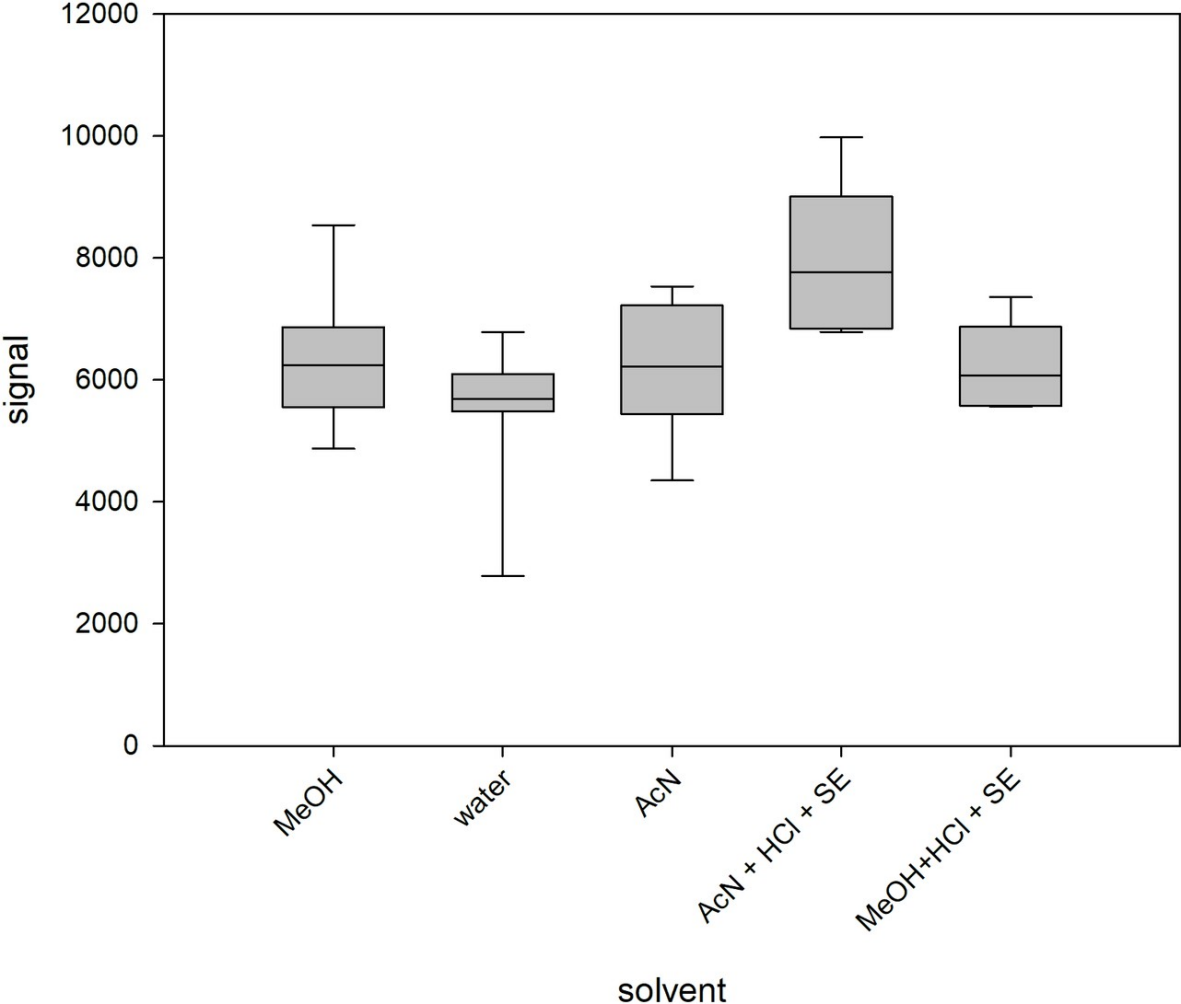
Signal intensity of BAC-C12 (100 μg/L) depending on solvent matrix (methanol; deionized water; acetonitrile [n = 8]; acetonitrile with HCl and Soil Extract (SE); methanol with HCl and SE [n = 6]).

The internal standard that was added immediately before the HPLC-MS/MS analysis should compensate possible matrix-effects on ionization and quantification of target analytes. In order to determine the magnitude of matrix-effects on the ionization and quantification of QAACs, we recorded the signal of a concentration of 100 μg L^-1^ of BAC-C12 in pure solvents that were used for the extractions (MeOH and AcN), in MQ-water and in spiked soil extracts. Fig 4 shows similar signal intensities in different matrices and slightly elevated signals in AcN soil extracts. Fig 4 also shows that the instrumental drift is higher than the matrix effect, which has to be corrected by the internal standard.

#### Precision and reproducibility

Retention times were well reproducible and only showed a maximum overall inter-day shift of 0.2 min, regardless of analyte. This shift did not affect the results, since the MS/MS confirmed analyte identification. However, the linearity of the calibration is a key factor in detection precision. In the concentration range of 2.5 μg L^-1^ to 300 μg L^-1^ for each QAAC analyte, the regression coefficient R^2^ was never below 0.996, with a median value of 0.998, see Table 5. The precision of the extraction process expressed as standard deviation of the QAAC concentrations recovered after three repeated extractions of spiked soil samples lies between 1.3% for ATMAC-C12 and 3.0% for DADMAC-C12 (Table 3). Stable retention times, R^2^ values for the linearity ≥ 0.996 and the standard deviation of analyte recoveries from spiked replicate samples are confirming a good reproducibility and precision of the method. Nevertheless, we recommend using the qualifier ions (Table 2B) to confirm the identity of the analytes for less well characterized samples.

#### Environmental samples

After establishing the analytical method, we analyzed the QAAC concentration in different samples: Sewage sludge samples from Germany and soil samples from a Mexican Vertisol (Tlahuelilpan, Mexico) and a German luvisol (Hungen. Germany), a short overview of the soil characteristics is shown in Table 7. Sewage sludge A and B were from the same wastewater treatment plant in Giessen (Hesse) but sludge A was freeze-dried, whereas sludge B was air-dried. Sewage sludge C was from Bad Nauheim (Hesse) and freeze-dried as well. All wastewater treatment plants use a mechanical primary treatment and a biological secondary treatment. The sewage sludges were all dewatered sludges. As shown in Table 7 we found QAACs in all of those samples. Each of the analyzed QAAC was found in sewage sludge B and C. Soils and sewage sludge samples contained mainly BACs and DADMACs and less ATMACs, with DADMAC-C10 and BAC-C12 being detected in greatest concentrations of 24000 μg kg^-1^ and 38600 among all QAACs. This corresponded to the large amounts of these compounds used in numerous applications. BAC-C12 is one of the main components of industrial BAC mixtures and DADMAC-C10 is the main component of DADMAC mixtures (≥ 90%). Both mixtures are used for the cleaning of machines and rooms in all production sectors [27].

**Table 7:** Soil characteristics of German Luvisol and Mexican Vertisol.

	Mexican Vertisol	German Luvisol
C_total_ [%]	2.2	1.4
N_total_ [%]	0.2	0.2
pH	7.1	6.8
Clay content [%]	44.6	28.9
Carbonate [%]	0.3	0.5

**Table 8:** QAAC concentrations of agricultural soil from Mexico and Germany and sewage sludges from Germany analyzed with the developed method.

	Sewage sludge A	Sewage sludge B	Sewage sludge C	Mexican Vertisol irrigated with untreated wastewater	German Luvisol ten days after application of sewage sludge (0–30 cm)
	[μg/kg]	[μg/kg]	[μg/kg]	[μg/kg]	[μg/kg]
ATMAC-C8	17.0	3.0	10.4	< LOD	1.1
ATMAC-C10	322	27.0	194	< LOD	1.3
ATMAC-C12	388	34.0	795	3.5	0.8
ATMAC-C14	58.0	7.0	167	< LOD	1.4
ATMAC-C16	439	26.0	2470	7.0	1.4
BAC-C8	14.0	2.0	11.2	3.8	1.8
BAC-C10	123	18.0	328	3.0	1.6
BAC-C12	4780	562	38600	80.6	6.9
BAC-C14	933	55.0	19940	22.4	2.5
BAC-C16	68.0	10.0	3203	3.2	1.7
BAC-C18	38.0	9.0	919	2.1	1.8
DADMAC-C8	2260	247	3710	5.2	< LOD
DADMAC-C10	2870	222	24000	3.7	4.8
DADMAC-C12	44.0	20.0	16.6	< LOD	1.6
DADMAC-C14	< LOD	< LOD	22.1	< LOD	2.4
DADMAC-C16	< LOD	2.0	162	< LOD	< LOQ; > LOD
Chlormequat	< LOD	< LOD	< LOD	2.3	< LOD
Benzethonium	18.0	9.0	62.7	3.0	1.1

## Conclusion

QAACs are challenging analytes due to their surfactant nature requiring a specific analytical method for reliable extraction and quantification. In this work we present a fast and robust method for the simultaneous extraction of ATMACs, BACs, DADMACs, benzethonium and chlormequat from soils and sewage sludge and the subsequent quantification of their concentrations using HPLC-MS/MS. As far as we know, there is no method for the analysis of both environmental matrices. The reduction to only two eluents in HPLC makes the proposed method easier to handle and less error-prone compared to previously reported method(s). The achieved LOQ and LOD are equal to or lower than those reported in the literature for QAACs in comparable matrices. The good recovery rates and excellent precision of the proposed method enable and justify its use for enlarging our data base on the distribution and concentration levels of QAACs in soils and sewage sludge. First results of QAAC concentrations in soil and sewage sludge samples emphasize the importance of these analyzes.

## Supporting information

S1 Fig SPE Recovery of four different Cartridges, Chromabond HLB, Oasis HLB, Chromabond C18 EC and Chromabin CN. The challenging homologues of DADMAC (C14, C16) had the lowest recovery, Chlormequat is fully eliminated.
(TIF)

S2 Fig Chromatogram of 20 analytes in concentrations of 200 μg L^-1^ in Acn. (TIF)

S3 Fig TIC-chromatogram of 20 analytes in concentrations of 200 μg L^-1^ in Acn. (TIF)

S4 Fig Extracts during the SPE cleanup step. (TIF)

S1 File (XLSX)
