# Supplementary figures and images for: A fast and robust method for the extraction and analysis of quaternary alkyl ammonium compounds from soil and sewage sludge
Source: PLoS One. 2020 Aug 4;15(8):e0237020. doi: 10.1371/journal.pone.0237020 (PMC7402506; doi:10.1371/journal.pone.0237020)

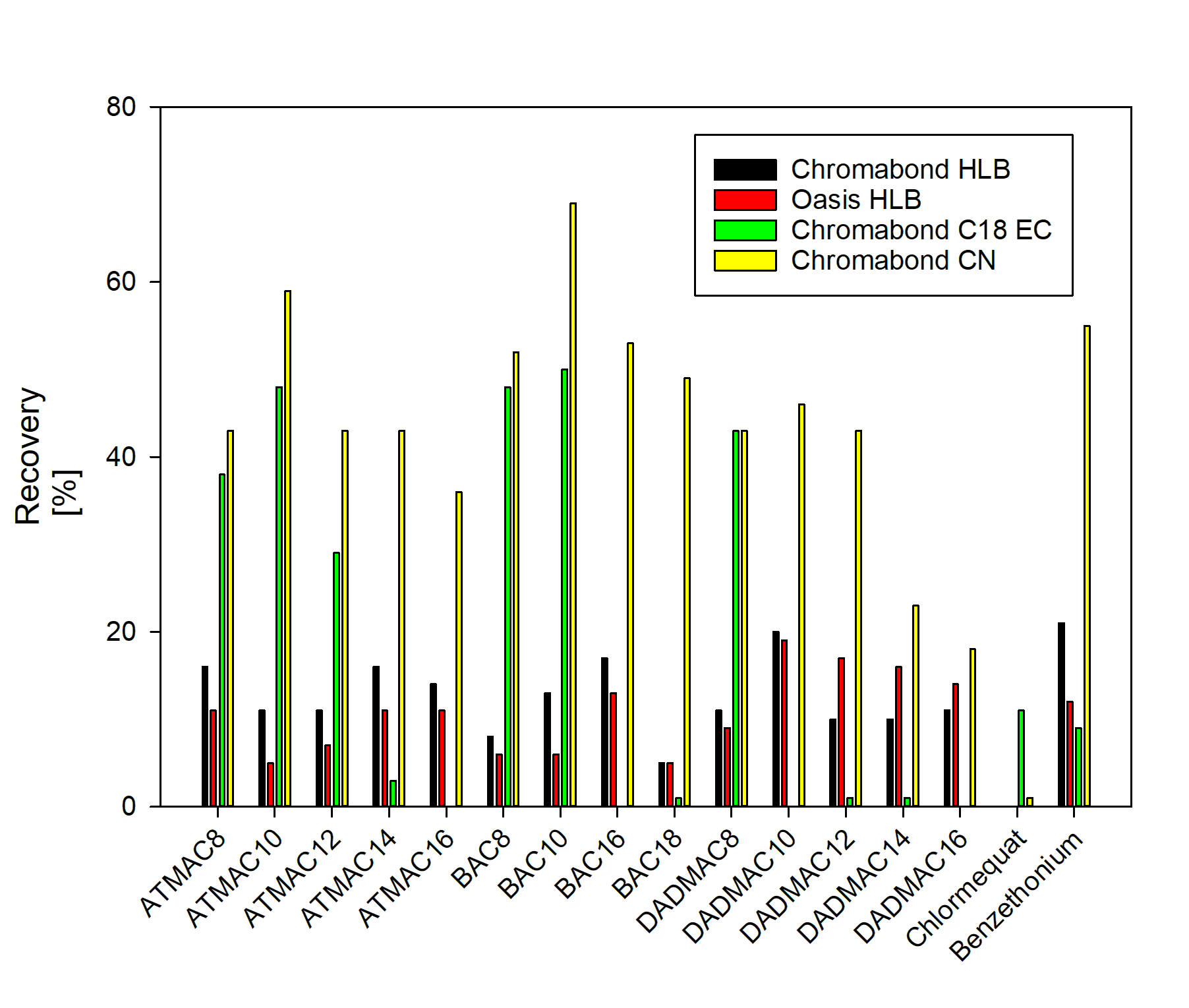

Supplement: S1 Fig — The challenging homologues of DADMAC (C14, C16) had the lowest recovery, Chlormequat is fully eliminated. (TIF) [file pone.0237020.s001.tif]

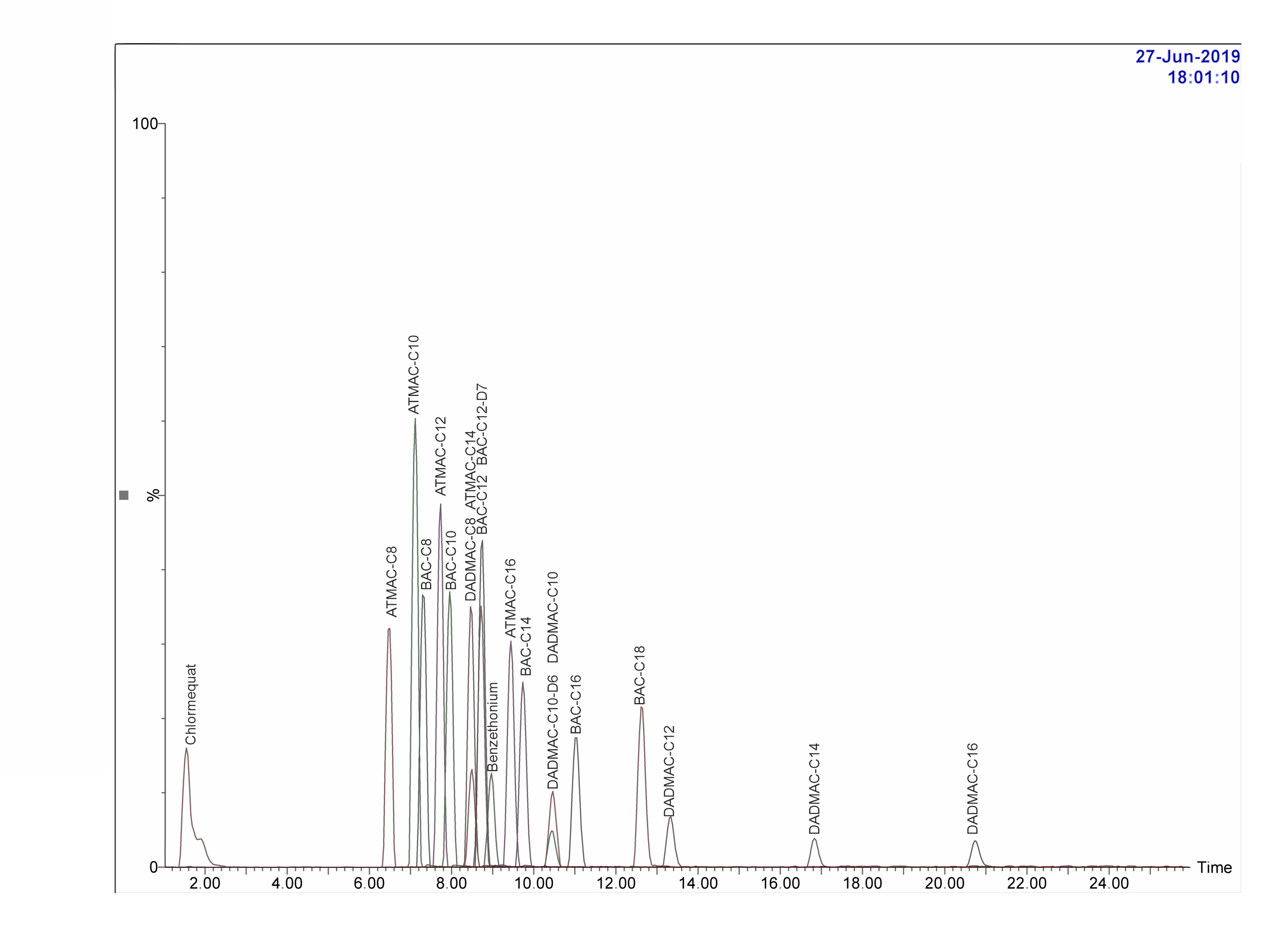

Supplement: S2 Fig — (TIF) [file pone.0237020.s002.tif]

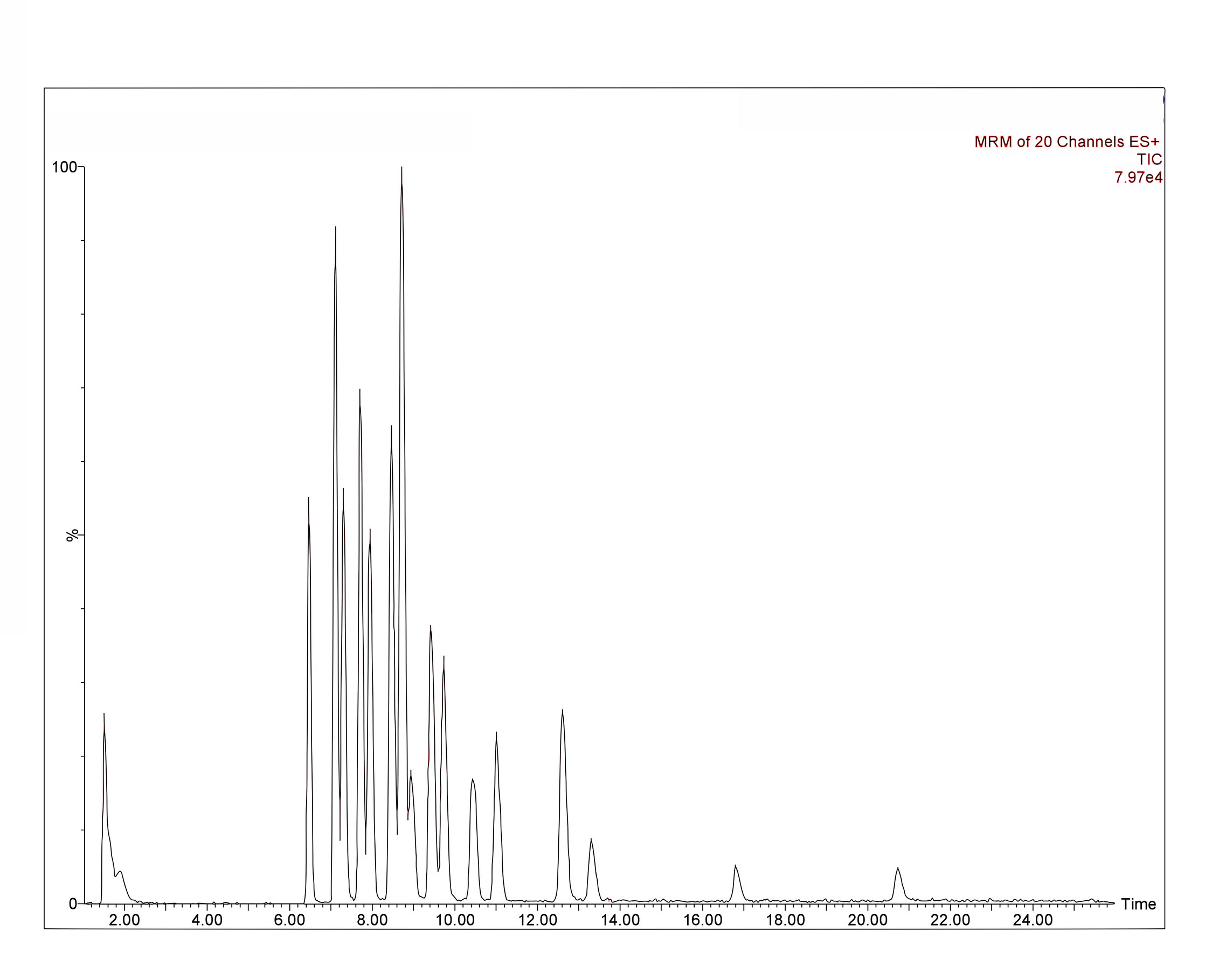

Supplement: S3 Fig — (TIF) [file pone.0237020.s003.tif]

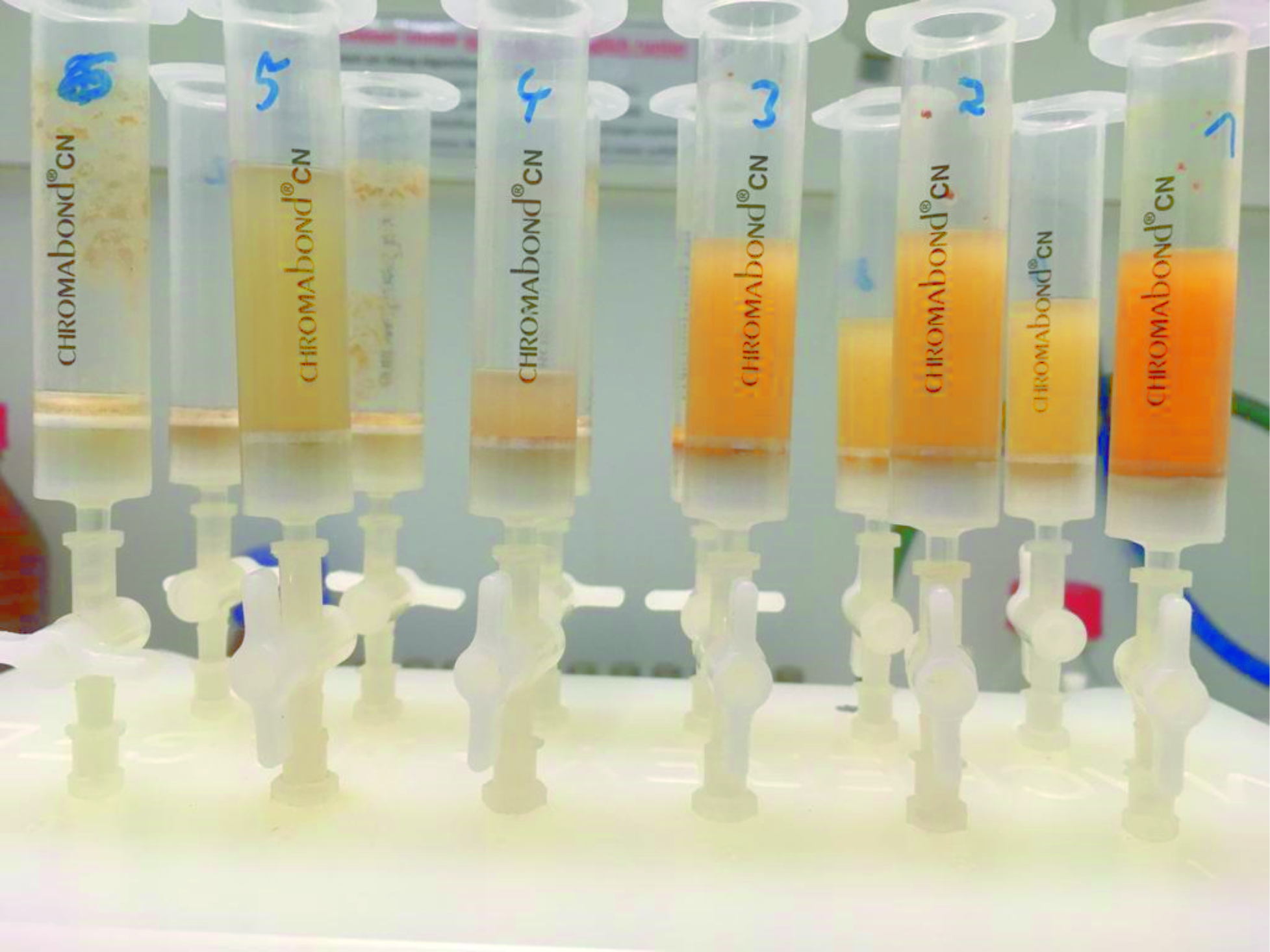

Supplement: S4 Fig — (TIF) [file pone.0237020.s004.tif]
